# Enhanced Luminous Efficacy and Stability of InP/ZnSeS/ZnS Quantum Dot-Embedded SBA-15 Mesoporous Particles for White Light-Emitting Diodes

**DOI:** 10.3390/nano12091554

**Published:** 2022-05-04

**Authors:** Chun-Feng Lai, Yu-Ching Chang, Yu-Shan Huang

**Affiliations:** Department of Photonics, Feng Chia University, Taichung 407, Taiwan; d0889838@o365.fcu.edu.tw (Y.-C.C.); d0963569@o365.fcu.edu.tw (Y.-S.H.)

**Keywords:** indium phosphide, quantum dots, SBA-15, thermal stability, mesoporous particle

## Abstract

Environmentally friendly quantum dots (QDs) of InP-based materials are widely investigated, but their reliability remains inadequate to realize their full potential and wide application. In this study, InP/ZnSeS/ZnS QDs (pristine QDs) were dispersed and embedded into Santa Barbara Amorphous-15 mesoporous particles (SBA-15 MPs) for the first time. A solvent-free method for preparing QD white light-emitting diodes (WLEDs) that is compatible with the WLED packaging process was developed. The photoluminescence (PL) spectrum of pristine QD powder exhibited cluster states and had huge redshift of approximately 23 nm. By comparison, the PL spectrum of the SBA-15 MP/QD hybrid powder had a slight redshift of approximately 8 nm, only because the pristine QDs were dispersed and embedded well in the SBA-15 MPs. The PL intensity of the SBA-15 MP/QD hybrid powder slightly decreased after heating and cooling compared with that of the pristine QDs. Moreover, the luminous efficacy of the SBA-15 MP/QD hybrid WLEDs was enhanced by approximately 14% compared with that of the pristine QD-WLEDs. Furthermore, reliability analysis revealed that the SBA-15 MPs could improve the stability of the pristine QDs on chips. Thus, these MPs promise good potential for applications in mini-LEDs in the future.

## 1. Introduction

The use of light-emitting diodes (LEDs) for lighting and display has rapidly increased, because LEDs are more energy efficient than traditional lighting lamps [1,2,3]. As novel materials for next-generation displays, quantum dots (QDs) have many advantages such as tuned wavelength, high efficacy, and color purity [4,5]. Cadmium (Cd)-based QDs have excellent photophysical properties. Unfortunately, the Cd element poses environmental concerns according to the specification of the European Restriction of Hazardous Substances (RoHS). By comparison, light-emitting materials for display applications based on indium phosphide (InP) are compliant with RoHS standards. Although InP-based QDs are suitable for commercial display applications, their crystallization behavior, optical properties, and stability are difficult to control synthetically [6,7]. Thus, the stability of InP-based QDs must be improved through other passivation structures.

Many researchers have recently demonstrated that InP-based QDs have a high photoluminescence quantum yield (PLQY) and a narrow full width at half maximum (FWHM). Hens et al. demonstrated that halide precursor and aminophosphine have different reaction rates, and they were able to obtain an 80% PLQY after shell passivation [8]. Moreover, the efficacy and stability of InP-based QDs can be improved using the ZnSeS gradient shell structure [9,10]. Several researchers have attempted to improve the color purity (narrow FWHM) of InP-based QDs [11,12,13]. InP-based QDs have been used to develop the PL layer of color-converter white light-emitting diodes (WLEDs) to control the chromatic properties of WLEDs. However, achieving high luminous efficacy is faced with some challenges, particularly the instability of QD-WLEDs. Another issue is that QD clusters strongly absorb the emitted light, leading to high reabsorption losses. Therefore, in QD-WLED packaging process, QD powder must be dissolved by an organic solvent and then dispersed into a transparent matrix to form QD/resin composites to prevent oxidation [4,5]. Unfortunately, the use of a solvent in dispersing QDs is incompatible with the WLED packaging process. Furthermore, QD-WLEDs that utilize a high QD concentration increase the ratio of light radiant power, but such a high QD concentration could cause high conversion loss because of the heavy reabsorption by the QD clusters [14]. Therefore, the loss in optical energy will increase the operating temperature and accelerate the life decay of InP-based QDs and compromise the reliability of InP-based QD-WLEDs.

Various mesoporous particles (MPs) are widely used to embed QDs inside themselves to improve their environmental stability and suppress reabsorption losses [15,16]. Compared with common mesoporous silica, the ordered hexagonal structure of Santa Barbara Amorphous-15 (SBA-15) MPs have more advantages such as a larger pore size (5–15 nm) and thicker walls (3–6 nm). As a result, these properties endow SBA-15 MPs with good thermal stability and chemical resistance [17,18]. The improved luminous efficacy of Cd-based QD-WLEDs is due to the waveguide effect of SBA-15 MPs, and this effect offers great potential in suppressing reabsorption losses and increasing QD light-conversion efficiency [19,20].

InP-based QDs have excellent performance, but their stability must still be improved if they are to be applied to QD-WLEDs. In this study, InP-based QDs were embedded into SBA-15 MPs for the first time. InP/ZnSeS/ZnS QDs (pristine QDs) were synthesized via a one-step hot-injection synthesis method. The pristine QDs were embedded into the SBA-15 MPs to improve the dispersion, thermal stability, and intensity of the PL spectra of pristine QDs. A solvent-free method for fabricating QD-WLEDs that is compatible with the WLED packaging process was developed. Finally, pristine QDs and SBA-15 MP/QD hybrid WLEDs were prepared and measured to study the influence of the SBA-15 MPs on their optical performance. The results showed that the SBA-15 MPs enhanced the luminous efficacy of the QD-WLEDs. Moreover, reliability analysis of the QD-WLEDs revealed that their luminous efficacy was in excess of 14%. Their high stability was beneficial in prolonging the lifetime of the SBA-15 MP/QD hybrid WLEDs by over 96 h compared with that of the pristine QD-WLEDs. Low-toxic and environmentally friendly luminescent materials used for lighting sources have always been a world goal.

## 2. Materials and Methods

### 2.1. Experimental Materials

Indium(III) iodide (InI_3_, 99.99%), zinc(II) bromide (ZnBr_2_, 98.0%), tris(diethylamino)phosphine ((DEA)_3_P, 97%), sulfur powder (S), selenium powder (Se, 99.99%), zinc stearate (Zn(St)_2_, 65%), trioctylphosphine (TOP, >97%), oleylamine (OLA, 90%), 1-octadecene (ODE, 99.5%), 1-dodecanethiol (DDT, >98%), hexane (99.9%), and ethyl alcohol (EtOH, 99.9%) were purchased from Sigma–Aldrich (St. Louis, MO, USA). Silicone (OE-7662) was bought from Dow Corning (Midland, MI, USA). SBA-15 (average pore size of approximately 11 nm) was procured from Shengsen Nano Tech., Co., Ltd., (Taichung, Taiwan). Blue LED devices (4014 plastic leaded chip carrier (PLCC)) were acquired from Lextar Electronics Co., Ltd., (Hsinchu, Taiwan).

### 2.2. Synthesis of InP/ZnSeS/ZnS QDs

**Scheme 1:**
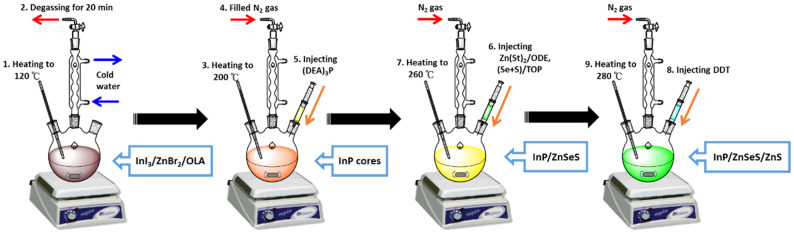
Schematic synthesis of green-emitting InP/ZnSeS/ZnS QDs.

As shown in Scheme 1, InP/ZnSeS/ZnS QDs (as pristine QDs) were synthesized via a one-step hot-injection method. First, InI_3_ (0.45 mmol) and ZnBr_2_ (2.925 mmol) were mixed with 5 mL of OLA in a 50 mL three-necked flask. Then, the mixture was heated to 120 °C under vacuum for 20 min, refilled with N_2_, and heated to 200 °C. Afterward, (DEA)_3_P (2.75 mmol) was quickly injected into the reaction mixture, which had been synthesized for 6 min. Subsequently, Zn(St)_2_ was dissolved in ODE and Se (0.3 mmol) and S (0.8 mmol) were dissolved in TOP, and these solutions were injected into the reaction mixture. The reaction mixture was heated to 260 °C for 120 min. Successively, 1.5 mL of DDT was injected by raising the temperature to 280 °C. The ZnS shell was grown by reacting it at 280 °C for 1 h. The ZnSeS and ZnS shells were grown in the same reaction flask. Finally, the resulting pristine QDs were precipitated and washed with EtOH.

### 2.3. Fabrication Method of SBA-15 MP/QD Hybrid Powder

**Scheme 2:**
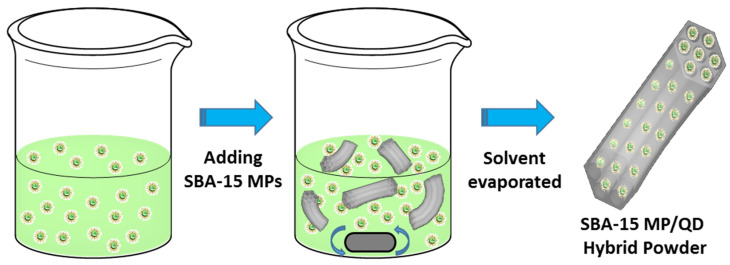
Schematic illustration of the formation of SBA-15 MP/QD hybrid powder.

As shown in Scheme 2, the pristine QDs embedded into the SBA-15 MPs (as SBA-15 MP/QD hybrid powder) were prepared via the adsorption method in hexane solution. First, the pristine QD powder was dispersed in hexane solution (5 mg/mL). The optimized mass ratio of the SBA-15 MPs (5:1) was added to the pristine QD solution [20]. The mixture solution was stirred to allow the SBA-15 MPs to physically adsorb the pristine QDs. Finally, the SBA-15 MP/QD hybrid powder was obtained when all the remaining hexane was completely evaporated.

### 2.4. Fabrication Method of SBA-15 MP/QD Hybrid WLEDs

The different mass ratios of the pristine QD powder and the SBA-15 MP/QD hybrid powder were mixed with the silicone matrix. Then, the SBA-15 MP/QD hybrid silicone mixtures were dispensed into a 4014 PLCC LED package containing blue InGaN LED (emission wavelength of approximately 450 nm) by an LED autodispenser machine. Finally, the white light-emitting pristine QD-WLEDs and the SBA-15 MP/QD hybrid WLED packages were thermally cured at 150 °C for 1 h under an oven equipped with nitrogen gas.

### 2.5. Characterizations

Field emission transmission electron microscope (FETEM) images were captured using a transmission electron microscope (JEM-2100F, JEOL, Tokyo, Japan). The foil preparation for TEM was observed as following. First, the QD nanoparticles were mixed with toluene solvent (0.1 mg/mL), then sonicated for few minutes. A carbon-coated grid was placed on paper, and a 1.0 μL of QD solutions was dropped onto the grid (three times) using a micropipette. Finally, it was kept dry under a vacuum oven that was thoroughly degassed at a reduced pressure of 80 °C for one day. The spectra of the pristine QD-WLEDs and the hybrid WLEDs were recorded using an energy-dispersive spectrometer (X-MaxN TSR, OXFORD, Oxford, UK). The Brunauer–Emmett–Teller (BET) surface areas and pore sizes of the SBA-15 MPs were measured by a high-resolution surface area and porosity analyzer (ASAP2020, Micromeritics, Norcross, GA, USA). Absorbance spectrum was measured by a UV–Vis spectrophotometer (UV-1900i, Shimadzu, Kyoto, Japan). PL spectra and PLQY were measured using a spectrofluorometer (FluoroMax-4, Horiba, Paris, France) with a 6-inch integrated sphere (Quanta-*ϕ*, Horiba). The PL lifetime of time-resolved PL (TRPL) was determined with a picosecond-pulsed UV laser (LDH-P-C-375B, PicoQuant, Berlin, Germany), and its dynamics were resolved using a time-correlated single-photon counting device (Timeharp 260 Nano, Picoquant, Berlin, Germany). The luminous flux, luminous efficacy, and International Commission on Illumination (CIE) color chromaticity coordinates (*x*, *y*) of the QD-WLEDs were measured using an integration sphere system (Labsphere, North Sutton, NH, USA).

## 3. Results

### 3.1. Optical Performances of InP/ZnSeS/ZnS QDs Solution

**Figure 1:**
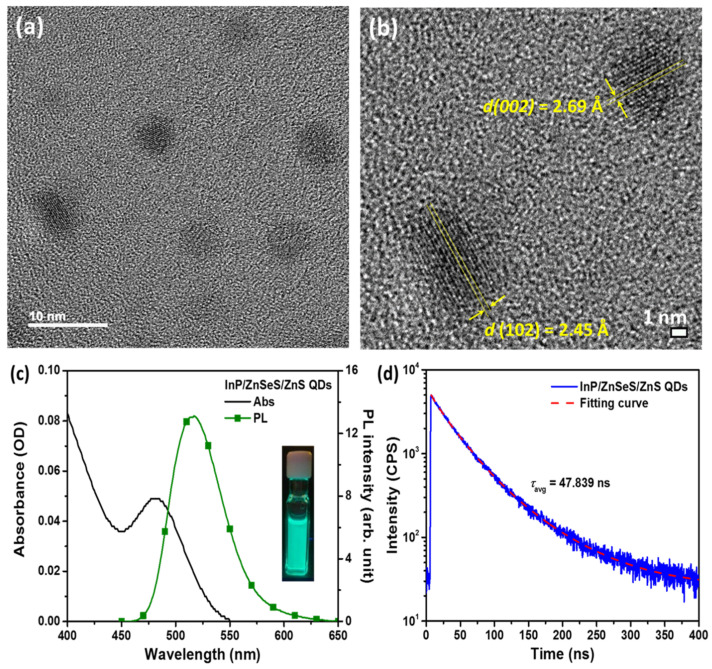
(**a**,**b**) FETEM image for synthesis pristine QDs; (**c**) UV–Visible absorbance and PL spectrum; (**d**) TRPL decay and the fitted curve of pristine QD solution. Inset (**c**) exhibits the sample solution under UV 365 nm light.

The FETEM image of the pristine QDs is given in Figure 1a. It shows that the pristine QDs were homogenously distributed with no agglomeration. The FETEM image and histogram of the particle size distribution of the pristine QDs are shown in Figure S1. The pristine QDs have a particle size distribution ranging from 5 to 8 nm. The average particle size (*d*_avg_) of the pristine QDs was measured as 6.35 ± 0.58 nm. Moreover, the FETEM presented an interlayer spacing of 2.45 and 2.69 Å for the pristine QDs, which were attributed to the (102) and (002) planes of the zinc blende structure, respectively [21,22]. The UV–Vis absorbance and PL spectra of the pristine QD solution were measured (Figure 1b). The 1st excited peak of the UV–Vis absorption and PL emission peaks of the pristine QDs at 481 and 517 nm, respectively, were ascribed to the electronic absorption and radiative recombination of excitons between the conduction band and the valence band under the QD dispersion state. Furthermore, the FWHM of the PL spectrum and the PLQY of the pristine QD solution was approximately 54 nm and 16%, respectively. The TRPL decay curve in Figure 1c was fitted with triple-exponential decay function, which exhibited a fast component *τ*_1_ (8.36 ns, *f*_1_ = 1.4%), a middle component *τ*_2_ (32.74 ns, *f*_2_ = 59.9%), and a longer component *τ*_3_ (75.34 ns, *f*_3_ = 38.7%). The relatively short-lived lifetime (*τ*_1_), middle-lived lifetime (*τ*_2_), and long-lived lifetime (*τ*_3_) are related to the radiative recombination channels of intrinsic excitons, interaction between excitons and surface traps, and interaction between excitons and surface defect-related components, respectively [5]. The average photon lifetime (*τ*_avg_) of the pristine QD solution states was approximately 47.84 ns.

### 3.2. Realization of QD-Embedded SBA-15 MPs

SBA-15 MPs with a two-dimensional hexagon pore size comparable to that of the pristine QDs were used because they exert a waveguide effect on the light emitted from the QDs [19,20]. The SBA-15 MPs had a rod-shaped geometry with a large aspect ratio (Figure S2). The FETEM images of the pores from the top-view and the lateral-view of the SBA-15 MPs are given in Figure S2b,c, respectively. The results of the BET measurement and analysis are provided in Figure S3. The pore-size distribution (PSD) was obtained by the Barrett–Joyner–Halenda (BJH) model from the desorption branch. The N_2_ adsorption–desorption isotherms of the SBA-15 MPs are shown in Figure S3a; the isotherms could be identified as type IV, indicating the presence of a mesoporous structure. As shown in Figure S3b, the PSD of the SBA-15 MPs ranged from 7 to 16 nm, which was mostly concentrated at 11.25 nm. The specific BET surface area and the total pore volume of the SBA-15 MPs were 510.54 m^2^/g and 1.11 cm^3^/g, respectively.

**Figure 2:**
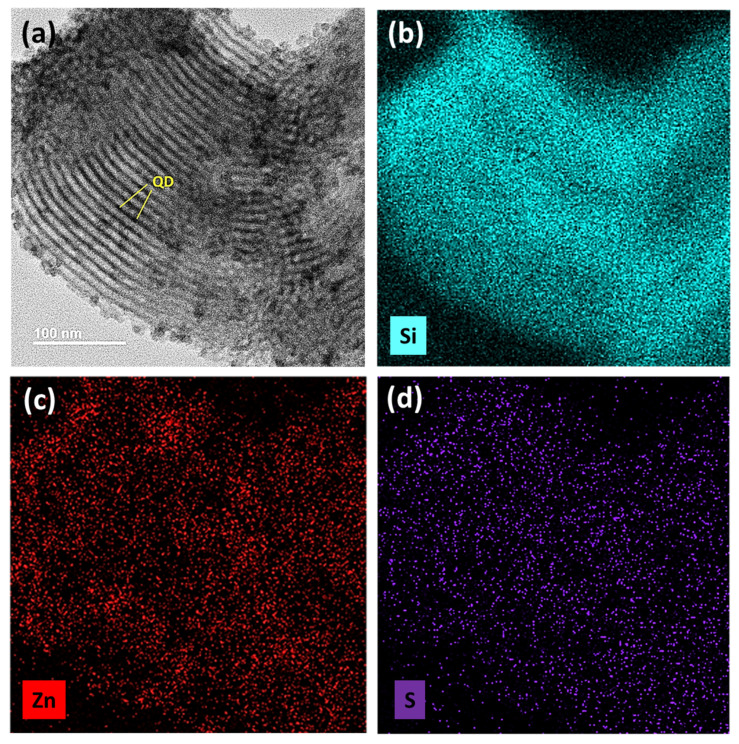
(**a**) The FETEM image of the SBA-15 MP/QD hybrid powder; (**b**–**d**) elemental Si, Zn, and S mapping images of the MP/QD hybrid powder, respectively, collected from the same location as (**a**).

The pristine QDs inside the SBA-15 MPs must be dispersed to suppress the reabsorption of the pristine QDs. A wet-mixing process of facile adsorption method was adopted to obtain the SBA-15 MP/QD hybrid composites. Notably, the SBA-15 MP/QD hybrid powder is entirely compatible with the current WLED packaging process that uses phosphorous powder (solvent free). As can be observed in Figure 2a, which shows the top-view FETEM image of the SBA-15 MP/QD hybrid powder, the pristine QDs were adsorbed inside the SBA-15 MPs. The elemental mapping images of Si, Zn, and S at the same location are given in Figure 2b–d The elements of the pristine QDs, such as S and Zn, could be evidently observed, indicating that these spherical pristine QDs were adsorbed inside the SBA-15 MPs. The SBA-15 MPs served as a good matrix for the dispersal of the pristine QDs. The pore size of the SBA-15 MPs was enough for the diameter of the pristine QDs. Most importantly, this pore size effectively helped in separating the pristine QDs by their wall and preventing the aggregation of the pristine QDs.

### 3.3. Optical Performances of SBA-15 MP/QD Hybrid Powder

**Figure 3:**
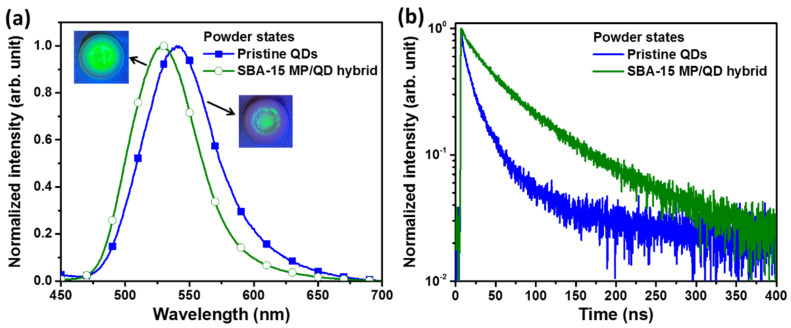
(**a**) PL spectra of pristine QD powder and SBA-15 MP/QD hybrid powder, and the inset shows their image under UV 365 nm; (**b**) TRPL decay profiles for pristine QD powder and MP/QD hybrid powder in air excitation with a 375 nm fs laser.

The PL spectrum of the pristine QD powder and that of the pristine QD solution was remarkably different (Figure 1b and Figure 3a). The PL spectrum of the pristine QD powder exhibited a large FWHM at approximately 66 nm, and its emission peak redshifted from 517 to 541 nm compared with that of the pristine QD solution, because the pristine QD powder was the agglomeration state. Considering that the Förster resonance energy transfer (FRET) of the pristine QD agglomeration caused the redshift of the emission peaks, our experimental results demonstrated that the use of SBA-15 MP/QD hybrid powder improved the aggregation of the of the pristine QDs (Figure 3a). The SBA-15 MP/QD hybrid powder showed a green emission peak at approximately 529 nm and an FWHM at approximately 60 nm of the PL spectrum. The PLQY of the pristine QD powder and the SBA-15 MP/QD hybrid powder was estimated as 8.0% and 25.5%, respectively. The improvement in PLQY suggested that the passivation of the pristine QDs by the SBA-15 MPs led to the suppression of the reabsorption behavior and an enhancement of the radiative recombination of excitons.

The TRPL of the pristine QD powder and the SBA-15 MP/QD hybrid powder was also compared. Their TRPL decay curves were characterized to investigate further the effects of the pristine QDs on the variation in photon lifetime through the SBA-15 MP mixture processes. The photon lifetime curves of the pristine QD powder and the SBA-15/MP hybrid powder for the exciton emission peak at approximately 541 and 529 nm, respectively, are presented in Figure 3b. The *τ*_avg_ of the pristine QD powder was approximately 52.6 ns, which was extracted using the triple-exponential decay equation fitting with three component lifetimes of *τ*_1_ (5.7 ns, 11.1%), *τ*_2_ (22.3 ns, 54.2%), and *τ*_3_ (115.2 ns, 34.7%). The TRPL decay curve of the SBA-15 MP/QD hybrid powder (*τ*_avg_ = 75.8 ns) in Figure 3b was fitted with a bi-exponential decay function, which exhibited a fast component (*τ*_a_ = 24.3 ns, *f*_1_ = 18.2%) attributed to the radiative recombination channels of intrinsic excitons and a longer component (*τ*_b_ = 87.2 ns, *f*_2_ = 81.8%) from the emission of trap states. The increase in the lifetime of the SBA-15 MP/QD hybrid powder compared with that of the pristine QD powder demonstrated that the embedding of the pristine QDs into the SBA-15 MPs had some advantages such as an increase in the recombination contribution of intrinsic excitons increased. This phenomenon might be due to the good dispersion provided by the SBA-15 MPs, which reduced the FRET of the pristine QDs and elongated their photon lifetime. Thus, the efficient usage of excited carriers was favored, resulting in a long photon lifetime.

**Figure 4:**
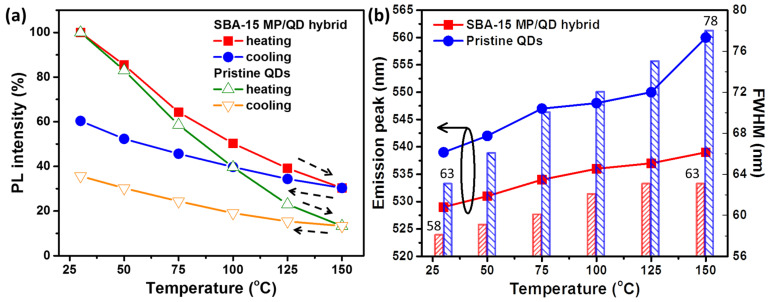
(**a**) Variations in the PL intensity ratio and the (**b**) emission peaks and FWHM of pristine QD powder and SBA-15 MP/QD hybrid powder during different measurement temperatures (the arrows in (**a**) indicate the testing temperature).

Thermal stability is another important factor for QD applications because of thermally assisted structural decomposition and defect trapping at high temperatures [5]. Nevertheless, surface coating can reduce the surface degradation of QDs and enhance their thermal stability. Silica shell acts as a buffer to prevent damage from thermal effects effectively. However, these effects may be associated with the dense structure of silica [5]. Therefore, the advantages of embedding the QDs into the SBA-15 MPs in ensuring thermal stability were explored and discussed herein. A high temperature (approximately 150 °C) was applied to investigate the thermal stability of all samples under atmospheric pressure. The superiority of the SBA-15 MP/QD hybrid powder over the pristine QDs against thermal annealing was highlighted by annealing the pristine QD powder and the SBA-15 MP/QD hybrid powder from 30 to 150 °C (Figure 4a). As the temperature increased to 150 °C, the relative PL intensity of the pristine QD powder and the SBA-15 MP/QD hybrid powder slowly decreased to 13.3% and 30.3%, respectively, which might have been caused by the thermal quenching effect. By contrast, in the case of the SBA-15 MP/QD hybrid powder, the thermal quenching effect was suppressed. After cooling, the percentage of the PL intensity decay of the pristine QD powder and the SBA-15 MP/QD hybrid powder was approximately 35.7% and 60.4%, respectively. The unrecoverable behavior of emission intensity might have been caused by the additional nonradiative recombination centers generated at high temperature [18]. The emission intensity of the pristine QD powder sharply decreased with increasing temperature. The thermal stability of the QDs was greatly improved after the addition of the SBA-15 MPs.

The SBA-15 MP/QD hybrid powder demonstrated better thermal stability even after severe PL quenching than the pristine QDs. Therefore, the SBA-15 MPs’ structures effectively protected the pristine QDs against thermal degeneration due to the high temperature. As a result, the SBA-15 MP/QD hybrid powder showed better and improved PL robustness against thermal stress than the pristine QDs. Moreover, the PL emission peak of the pristine QD powder and the SBA-15 MP/QD hybrid powder exhibited an apparent redshift at approximately 21 and 7 nm, respectively, from 30 to 150 °C (Figure 4b). The FWHM of the pristine QD powder substantially increased at approximately 15 nm variations from 30 to 150 °C. Furthermore, the FWHM of the SBA-15 MP/QD hybrid powder increased by approximately 5 nm (Figure 4b). Hence, the synthesized SBA-15 MP/QD hybrid powder exhibited excellent thermal stability. The data above prove that the thermal stability of the SBA-15 MP/QD hybrid powder was better than that of the pristine QDs. In addition, the InP/ZnS core/shell QD film demonstrated the enhancement of optical properties through rapid thermal annealing (RTA) [23]. Therefore, the pristine QD powder and SBA-15 MP/QD hybrid powder were also treated via RTA in this study; the temperature was set at 150 ℃ for a treatment time of 5 min. The PL spectra of two samples for untreated and treatment are displayed in Figure S4a,b), respectively. After annealing at 150 °C, the PL intensities of the PL spectra and PLQY for two samples decreased. The intensity of the emission decreased through RTA, which may be that the annealing process introduced other nonradiative recombination centers, suppressing the free carrier emission. Finally, the SBA-15 MP/QD hybrid powder through high-temperature treatment was still better than that of the pristine QD powder.

### 3.4. Optical Performances of SBA-15 MP/QD Hybrid LEDs

InP-based QDs with low toxicity, high QYs, and broad color tunability are particularly suitable in constructing WLEDs for indoor/outdoor illumination and display backlighting [24,25,26,27]. In general, InP QDs can be applied in WLEDs as a PL emission layer. QD–resin composites, including pristine QD powder, and SBA-15 MP/QD hybrid powder as phosphors are directly dispensed on a blue InGaN LED to fabricate QD-WLED devices. Notably, the solvent-free method for fabricating QD-WLEDs is entirely compatible with the current WLED packaging process.

**Figure 5:**
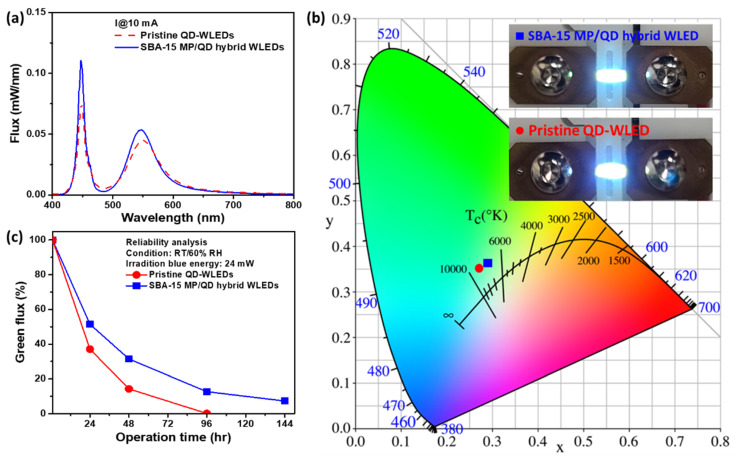
(**a**) EL spectra; (**b**) CIE diagram; (**c**) RA of the two types of QD-WLEDs subjected to RT/60% RH at an irradiation blue energy of 24 mW. Inset shows their image under current 10 mA.

In this study, the optical performance of QD-WLEDs were tested using an integrating sphere system from Labsphere. The injection current of the WLEDs was kept at 10 mA (injection electrical power of 14 mW). The luminous flux, CIE 1931 color coordinates, and luminous efficacy of the two types of QD-WLEDs were evaluated using the aforementioned integrating sphere system. Figure 5a shows the electroluminescence (EL) spectra of the prepared QD-WLEDs, which were measured under current at approximately 10 mA. Under an operating current of 10 mA, the CIE 1931 color coordinates of the pristine QDs and the SBA-15 MP/QD hybrid WLEDs were (0.27, 0.35) and (0.29, 0.36), respectively, as shown in Figure 5b. The pristine QDs and the SBA-15 MP/QD hybrid WLEDs exhibited a luminous efficiency of 74.8 and 85.4 lm/W, respectively. Therefore, the luminous efficacy of the SBA-15 MP/QD hybrid WLEDs was enhanced by approximately 14% compared with that of the pristine QD-WLEDs under similar color coordinates. Moreover, all QD samples of the QD-WLEDs were placed in an environment at room temperature with a relative humidity as high as 60% to confirm further the synergistic effect of light, water, and thermal stability on reliability analysis (RA). The two-type QD-WLEDs were continuously irradiated with 24 mW of blue energy (current under 10 mA) under RA measurement. As shown in Figure 5c, the SBA-15 MP/QD hybrid WLEDs showed a slower green flux decay rate than the pristine QD-WLEDs, confirming that their light, water, and heat stability were due to the SBA-15 MPs’ protection. After 144 h, the green flux of the pristine QD-WLEDs had fully decayed, whereas that of the SBA-15 MP/QD hybrid WLEDs maintained a 7.3% green flux of the initial value. The pristine QDs were protected by the SBA-15 MPs against photo-oxidation and thermal degradation even under high-energy irradiation and high temperature of the LED chip. These results demonstrated that the pristine QDs embedded into the SBA-15 MPs could enhance the luminous efficacy and stability of QD-WLEDs for QD-on-chip applications. This study also further promoted the progress in the application of SBA-15 MP/QD hybrid WLEDs in many fields such as solid-state lightings and displays.

## 4. Conclusions

In this study, we developed a solvent-free method for preparing QD-WLEDs by using SBA-15 MPs that is compatible with the WLED packaging process that uses phosphor powder. Compared with that of the pristine QD-WLEDs, the luminous efficacy of the SBA-15 MP/QD hybrid WLEDs was enhanced by approximately 14%. Furthermore, the embedding of the pristine QDs in the SBA-15 MPs adequately and simultaneously enhanced their stability against a water medium, high-energy blue light, high temperature of the LED chip, and harsh environment of oxygen/moisture according to RA. The RA results of the SBA-15 MP/QD hybrid powder demonstrated its better thermal stability and superior air stability than the pristine QD powder. InP-based QDs embedded on SBA-15 MPs will contribute to the improvement in the stability of QD-on-chip WLEDs for use in next-generation mini-LED and micro-LED applications.

## Data Availability

Data sharing is not applicable to this article.

## Supplementary Materials

The following supporting information can be downloaded at: https://www.mdpi.com/article/10.3390/nano12091554/s1, Figure S1: FETEM image and histograms of particle size distribution of InP/ZnSeS/ZnS QDs; Figure S2: The FETEM images of (a) SBA-15 MP and the pores of SBA-15 observed from (b) the top-view and (c) the lateral-view, respectively; Figure S3: (a) N_2_ adsorption–desorption isotherms and (b) pore-size distribution of SBA-15 MPs; Figure S4: PL spectra of the untreated (a) Pristine QD powder and (b) SBA-15 MP/QD hybrid powder and after annealing at 150 ℃ for 5 min at room temperature.
