# Supplementary material for: Enhanced Luminous Efficacy and Stability of InP/ZnSeS/ZnS Quantum Dot-Embedded SBA-15 Mesoporous Particles for White Light-Emitting Diodes
Source: Nanomaterials (Basel). 2022 May 4;12(9):1554. doi: 10.3390/nano12091554 (PMC9100065; doi:10.3390/nano12091554)
Supplement: Supplementary file 1 [file nanomaterials-12-01554-s001.zip › nanomaterials-1707460-supplementary.pdf]

nanomaterials

Supporting Information

Enhanced Luminous Efficacy and Stability of InP/ZnSeS/ZnS Quantum Dot-Embedded SBA-15 Mesoporous Particles for White Light-Emitting Diodes

Chun-Feng Lai *, Yu-Ching Chang and Yu-Shan Huang

Department of Photonics, Feng Chia University, Taichung 407, Taiwan; d0889838@o365.fcu.edu.tw (Y.-C.C.); d0963569@o365.fcu.edu.tw (Y.-S.H.)
* Correspondence: chunflai@fcu.edu.tw; Tel.: +886-4-24517250

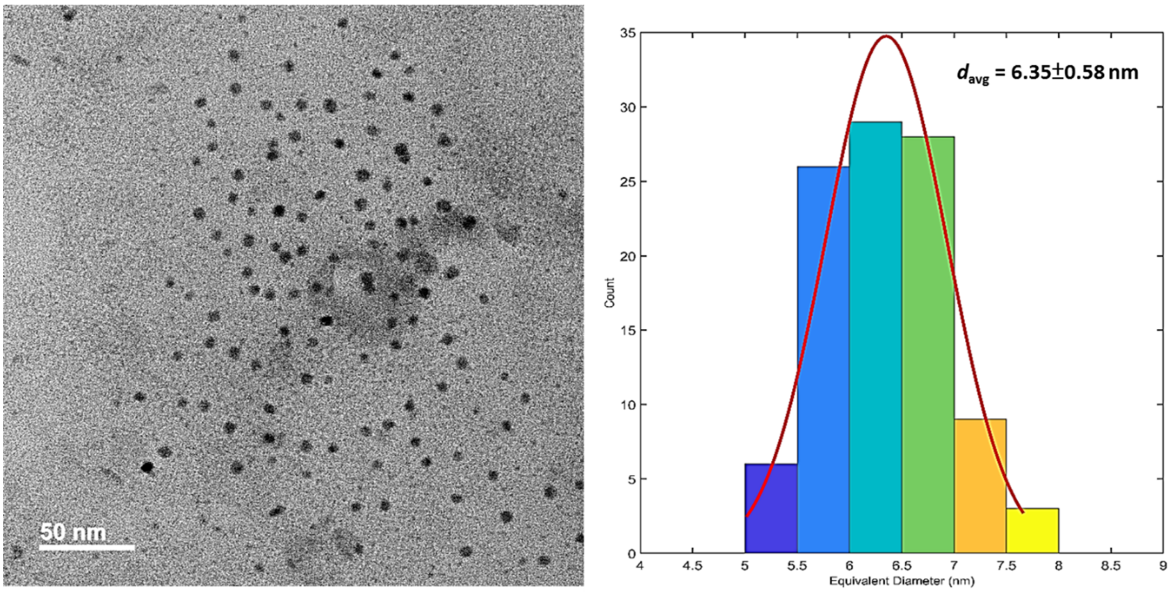

Figure S1. (**left**) FETEM image and (**right**) histograms of particle size distribution of InP/ZnSeS/ZnS QDs.

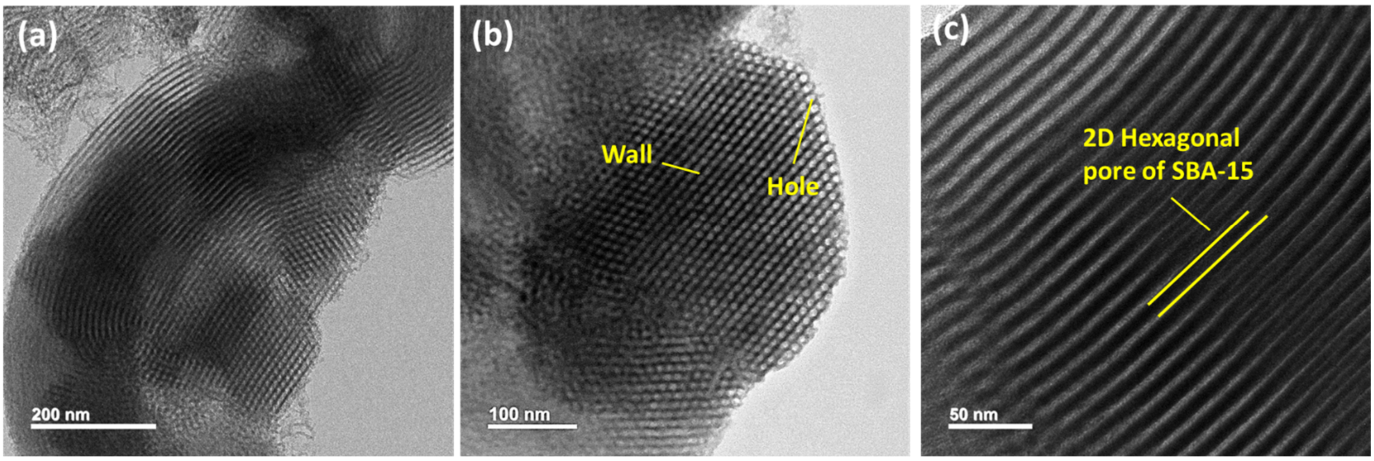

Figure S2. The FETEM images of (**a**) SBA-15 MP and pores of SBA-15 observed from (**b**) the top view and (**c**) the lateral view, respectively.

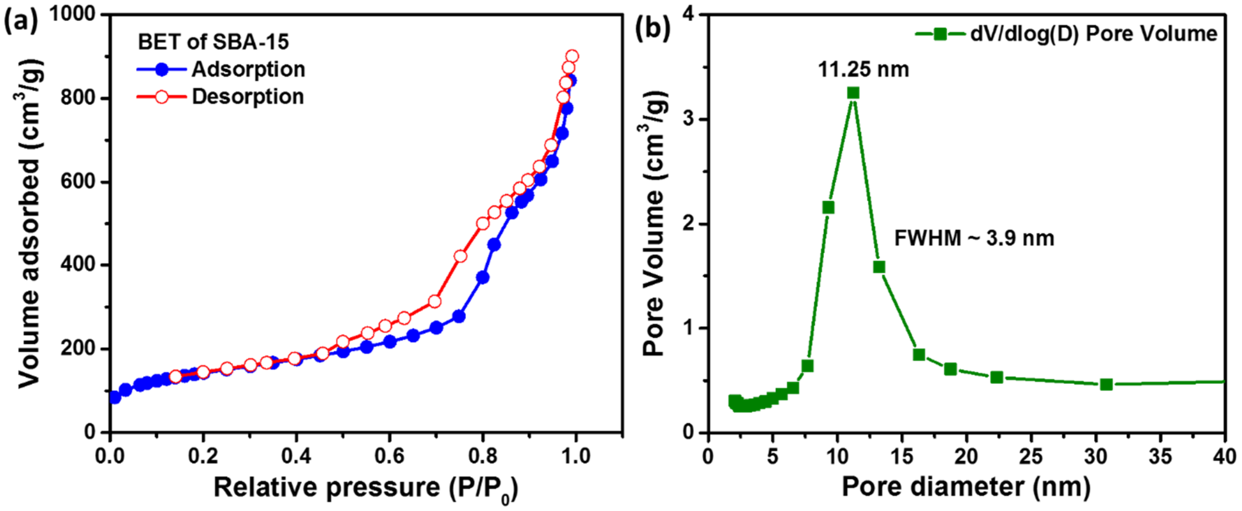

Figure S3. (**a**) N_2 adsorption-desorption isotherms and (**b**) pore size distribution of SBA-15 MPs.

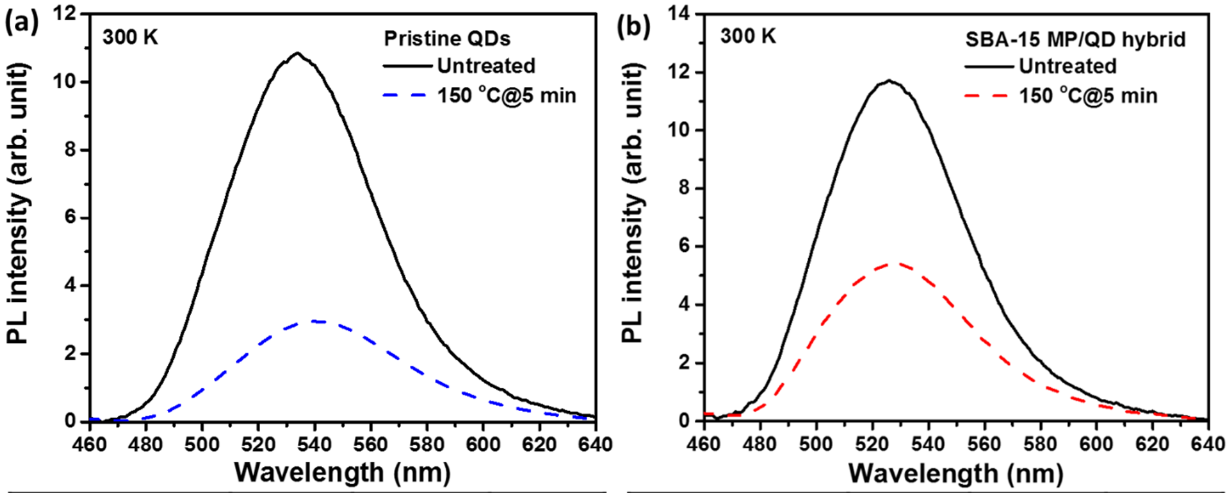

Pristine QDs	Peak (nm)	FWHM (nm)	PLQY (%)
Untreated	534	60.5	8.0%
150 ℃@5 min	542	67.2	2.9%

SBA-15 MP/QD hybrid	Peak (nm)	FWHM (nm)	PLQY (%)
Untreated	526	56.6	25.5%
150 ℃@5 min	528	60.0	19.3%

Figure S4. PL spectra of the untreated (**a**) Pristine QD powder and (**b**) SBA-15 MP/QD hybrid powder and after annealing at 150 ℃ for 5 min at room temperature.
